# First Contiguous Genome Assembly of Japanese Lady Bell (*Adenophora triphylla*) and Insights into Development of Different Leaf Types

**DOI:** 10.3390/genes15010058

**Published:** 2023-12-30

**Authors:** Ji-Nam Kang, Si-Myung Lee, Ji-Weon Choi, Seung-Sik Lee, Chang-Kug Kim

**Affiliations:** 1 Genomics Division, National Institute of Agricultural Sciences, Jeonju 54874, Republic of Korea; greatnami@korea.kr (J.-N.K.); tataby@korea.kr (S.-M.L.); 2 Postharvest Technology Division, National Institute of Horticultural and Herbal Science, Wanju 55365, Republic of Korea; jwcnpri@korea.kr; 3 Advanced Radiation Technology Institute, Korea Atomic Energy Research Institute, Jeongeup 56212, Republic of Korea; sslee@kaeri.re.kr; 4 Department of Radiation Science, University of Science and Technology, Daejeon 34113, Republic of Korea

**Keywords:** *Adenophora triphylla*, genome assembly, different leaf types, triterpenoid saponins

## Abstract

*Adenophora triphylla* is an important medicinal and food plant found in East Asia. This plant is rich in secondary metabolites such as triterpenoid saponin, and its leaves can develop into different types, such as round and linear, depending on the origin of germination even within the same species. Despite this, few studies have comprehensively characterized the development processes of different leaf types and triterpenoid saponin pathways in this plant. Herein, we provide the first report of a high-quality genome assembly of *A. triphylla* based on a combination of Oxford Nanopore Technologies and Illumina sequencing methods. Its genome size was estimated to be 2.6 Gb, and the assembled genome finalized as 2.48 Gb, containing 57,729 protein-coding genes. Genome completeness was assessed as 95.6% using the Benchmarking Universal Single-Copy Orthologs score. The evolutionary divergence of *A. triphylla* was investigated using the genomes of five plant species, including two other species in the Campanulaceae family. The species *A. triphylla* diverged approximately 51-118 million years ago from the other four plants, and 579 expanded/contracted gene families were clustered in the Gene Ontology terms. The expansion of the *β-amyrin synthase* (*bAS*) gene, a key enzyme in the triterpenoid saponin pathway, was identified in the *A. triphylla* genome. Furthermore, transcriptome analysis of the two leaf types revealed differences in the activity of starch, sucrose, unsaturated fatty acid pathways, and oxidoreductase enzymes. The heat and endoplasmic reticulum pathways related to plant stress were active in the development of round type leaf, while an enhancement of pyrimidine metabolism related to cell development was confirmed in the development of the linear type leaf. This study provides insight into the evolution of *bAS* genes and the development of different leaf types in *A. triphylla*.

## 1. Introduction

The Japanese lady bell (*Adenophora triphylla* var. *japonica*) is a perennial herb mainly distributed in China, Japan, Korea, and Russia [1]. *A. triphylla* belongs to the Campanulaceae family and grows in mountainous areas with good drainage [2]. It is an important medicinal plant used in oriental medicine to control symptoms of lung diseases, such as cough, sputum, and asthma [3]. Its roots contain different phytochemicals, such as saponin, inulin, polysthicol, triphyllol, and lupenone [4]. The plant’s leaves are used as a food source to prevent obesity in traditional Korean recipes [5]. In recent years, anti-inflammation, antitumor, and antidiabetic activities have been reported in *A. triphylla* [6], and their consumption has increased in oriental medicine, food, and health products [7].

The genome assemblies of two species, balloon flower (*Platycodon grandiflorus*) [8,9] and lance asiabell (*Codonopsis lanceolate*) [10], belonging to the Campanulaceae family, have been previously reported. However, the genome assembly of Japanese lady bell has not yet been reported. Genomic information can be used to guide breeding strategies for the cultivation and enhancement of crop quality. Next-generation sequencing (NGS) technologies have enabled accurate genome assembly [11], and highly heterozygous genomes have been assembled using a combination of short-read Illumina and long-read Oxford Nanopore Technologies (ONT) sequences [12].

Plant leaves are important organs that participate in photosynthesis, respiration, and transpiration [13], and have evolved many mechanisms to optimize leaf function according to their surrounding conditions [14]. Development of different leaf types is controlled through complex gene regulatory networks. Leaf evolution appears to depend on the architecture of cis-regulatory elements and the epigenetic states of responsive genes [15]. The complexity of leaf morphology during leaf development is generally related to the regulatory mechanism of various genes, including the transcription factor Knotted-like tale homeobox (KNOX1) [16].

Leaves of *A. triphylla* exhibit a wide range of morphological variations within the same species. They can be classified according to the types of leaves into lanceolate, cuneate, elliptic, and ovate forms [17]. However, in *A. triphylla*, few studies have reported on the biological processes involved in the development of different leaf types [6]. Most studies have focused on its classification, phytochemical functions, and nutritional composition [18,19,20]. Triterpenoid saponins are recognized as significant secondary metabolites in *A. triphylla*. Triterpenoid saponins have a wide range of pharmacological bioactivities, such as antihyperglycemic, anti-inflammation, antitumor, antioxidant, hemostatic, and hormone-like activities [21,22]. Triterpenoid saponins are synthesized via the mevalonic acid and methylerythritol 4-phosphate pathways [23]. In the Campanulaceae family, triterpenoid saponin pathways have been studied with diverse skeleton structures in α-amyrin, β-amyrin, dammarenediol, and lupeol metabolism [24]. In particular, *β-amyrin synthase* (*bAS*) gene families are key players in triterpenoid saponin biosynthesis [25]. However, triterpenoid saponin pathways in *A. triphylla* are not well characterized [26].

In this study, we report the first assembly of the *A. triphylla* genome. Furthermore, this study provides insight into the biological processes involved in the development of different leaf types and the evolution of triterpenoid saponin biosynthesis in *A. triphylla*.

## 2. Results and Discussion

### 2.1. Estimation of Genome Size

The genome size of *A. triphylla* was estimated using Jellyfish and GenomeScope. In total, 119.7 Gb of trimmed Illumina sequence data was obtained, and distributions of *k*-mer coverage based on Jellyfish analysis presented double peaks (Supplementary Figure S1). Using GenomeScope with optimal 17–23 *k*-mer values, we estimated that the haploid genome size ranged from 2.60–2.64 Gb with 2.32% heterozygosity (Supplementary Table S1). These results suggest that the *A. triphylla* genome is likely to be highly heterozygous and diploid. However, studies on the chromosome number in various *Adenophora* species have revealed that these species possess either 17, 34, or 51 haploids, indicating the potential presence of a tetraploid or hexaploid genome [27,28]. The *A. triphylla* genome is likely tetraploid, containing two similar subgenomes. This possibility may also be related to the high heterozygosity (2.32%) of the *A. triphylla* genome. The heterozygosity of autotetraploid plant genomes such as potato (*Solanum tuberosum*) is 6.8–7.9% (http://qb.cshl.edu/genomescope/genomescope2.0/analysis.php?code=example10, accessed on 14 December 2023) while the heterozygosity of allotetraploid genomes such as cotton (*Gossypium barbadense*) can be as high as 11% (http://qb.cshl.edu/genomescope/genomescope2.0/analysis.php?code=example8, accessed on 14 December 2023). However, heterozygosity levels can be high even in diploid plants. The heterozygosity of diploid plant genomes such as pear (*Pyrus pyrifolia*) is about 1.6% (http://qb.cshl.edu/genomescope/genomescope2.0/analysis.php?code=example3, accessed on 14 December 2023). These results suggest that heterozygosity levels vary in plant genomes and that high heterozygosity levels are not necessarily associated with plant polyploidy. Cytogenetic and genomic studies are very poor in *A. triphylla*. Therefore, it is necessary to confirm the chromosome number and ploidy level of this plant species through additional research in the future, referring to the genome sequence information obtained in this study.

### 2.2. Genome Assembly

To produce a high-quality genome sequence, we generated trimmed 123.6 Gb of Nanopore long-read and 119.7 Gb of Illumina short-read sequences (Supplementary Table S2). Two assembly pipelines, Pipeline 1 and Pipeline 2, were used to assemble the A. triphylla genome. Among the two sets generated by the two pipelines, the set produced by Pipeline 2 exhibited better quality than the one produced by Pipeline 1, as judged by the total assembly length, N50, and N90 (Supplementary Table S3). Therefore, the contig set generated by Pipeline 2 was selected as the final genome assembly sequence of A. triphylla and then used for further analysis. A total of 99.4% of the trimmed PE reads were mapped to genome sequences, and the mapped reads covered a maximum of 86.5% of the genome sequences. The long terminal repeat (LTR) assembly index (LAI) value [29] for assembly quality assessment of repetitive sequences was calculated as 11.8 (Supplementary Table S4). Finally, the quality of the assembled genome was evaluated using the Benchmarking Universal Single-Copy Orthologs (BUSCO) score. In the BUSCO analysis, 95.6% of genes were assembled in a complete form, while 1.0% were assembled in a fragmented form. The completeness of the genome was comparable with that of four genomes reported from C. lanceolata and P. grandifloras belonging to the Campanulaceae family (Supplementary Table S5) [8,9,10,25]. The genome was completed at 2.48 Gb, comprising 8432 contigs with an N50 value of 403.7 Kb (Table 1).

The pipeline based on NextDenovo showed higher assembly completeness metrics compared to assembly using Canu Pipeline in this study (Supplementary Table S3). These results indicate that NextDenovo can be efficient when assembling large-sized genomes with high heterozygosity such as the *A. triphylla* genome. In fact, NextDenovo improves long-read assembly in a variety of organisms. However, genome assembly of long reads must be appropriately selected, taking into consideration factors such as heterozygosity of the target organism, sequencing depth, convenience, cost, and computing resources. A variety of assemblers have been developed for genome assembly of long reads, and a perfect platform still does not exist [30,31].

### 2.3. Genome Annotation

A total of 57,729 protein-coding genes were predicted using the transcriptome data and protein sequences. The total length of the protein-coding genes was 55.3 Mb, with an average length of 958 bp and GC content of 43.6% (Table 1). Genome annotation identified 1.96 Gb of repeat sequences in *A. triphylla*, accounting for 79.2% of the genome. The most repetitive category was LTR retrotransposons, including *Gypsy* (35.0%) and *Copia* (13.4%) (Supplementary Table S6). In plants, *Gypsy*- and *Copia*-like retroelements are the major components that affect genetic and phenotypic variations [32].

Among the protein-coding genes, 88.4% were functionally annotated after comparing their homology against five known protein libraries: NCBI GenBank, *Arabidopsis thaliana* (Araport11) proteins, protein domains, and Gene Ontology (GO) and Kyoto Encyclopedia of Genes and Genomes (KEGG) databases. In total, 87.4% of the genes showed high similarity with known protein sequences deposited in the NCBI GenBank database and 65.9% had known conserved protein domains. A total of 45.4% of the genes were assigned to at least one GO term and 33.8% assigned to a pathway registered in the KEGG database (Supplementary Table S7). Features of this gene set were similar to those of previously reported genomes of species in the Campanulaceae family (Supplementary Table S8).

**Figure 1:**
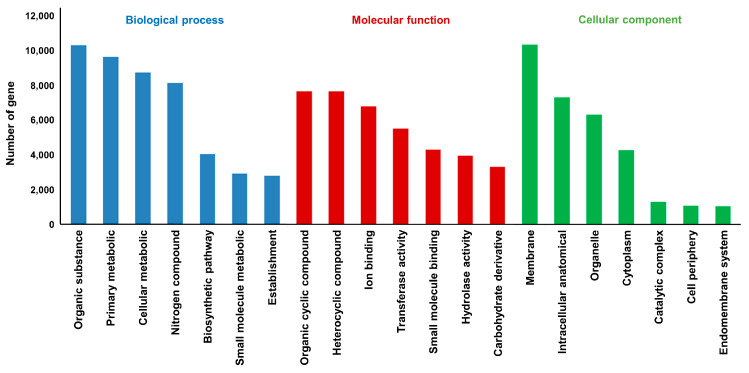
Gene histogram of Gene Ontology (GO) biological analysis in *Adenophora triphylla*. Colors represent the three GO categories: blue (biological process), red (molecular function), and green (cellular component). Height of the histogram bars represents the number of genes in the corresponding color sections at level three.

For functional classification of the annotated genes, 26,212 GO term genes were assigned to three functional categories: biological process (16,273), molecular function (20,310), and cellular component (15,504). Within the three categories at level three, the most abundant genes were assigned to organic substance metabolic processes, organic cyclic compound binding, and membrane processes (Figure 1).

### 2.4. Genome Comparison

**Figure 2:**
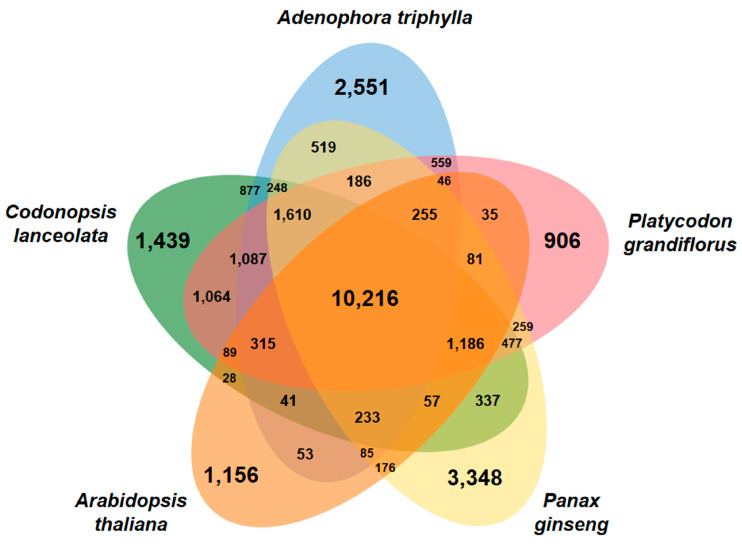
Gene distribution between *Adenophora triphylla* and four related species. Venn diagram showing the protein-coding genes sequenced among *A. triphylla*, *Codonopsis lanceolata*, *Platycodon grandiflorus*, *Panax ginseng*, and *Arabidopsis thaliana*. The numbers in overlapped regions indicate shared genes and those in non-overlapped regions the unique genes.

To determine the evolutionary relationship between *A. triphylla* and four other whole plant genomes (*C. lanceolata*, *P. grandiflorus*, *Panax ginseng*, and *Arabidopsis thaliana*) [10,25,33,34], we performed similarity-based gene clustering analysis. The genomes of *A. thaliana* and *P. ginseng* were added to investigate the evolutionary divergence of plants belonging to the Campanulaceae family (Supplementary Table S9). Gene clustering revealed that *A. triphylla* had 2551 unique gene clusters and shared 10,216 among the five genomes. Additionally, *A. triphylla* shared 877, 559, and 519 gene clusters with *C. lanceolata*, *P. grandiflorus*, and *P. ginseng*, respectively (Figure 2).

Using unique and shared gene clusters, GO enrichment analysis was performed to identify enriched functional categories. In *A. triphylla*, a total of 2551 unique and 10,216 shared genes were not enriched in any abundant GO terms. However, 877 shared genes between *A. triphylla* and *C. lanceolate* were mostly clustered as GO terms related to the translation process (Supplementary Table S10). The 559 genes shared with *P. grandiflorus* were the most clustered in defense response GO terms (Supplementary Table S11), and the 519 genes shared with *P. ginseng* mostly clustered in microtubule-based movement GO terms (Supplementary Table S12).

**Figure 3:**
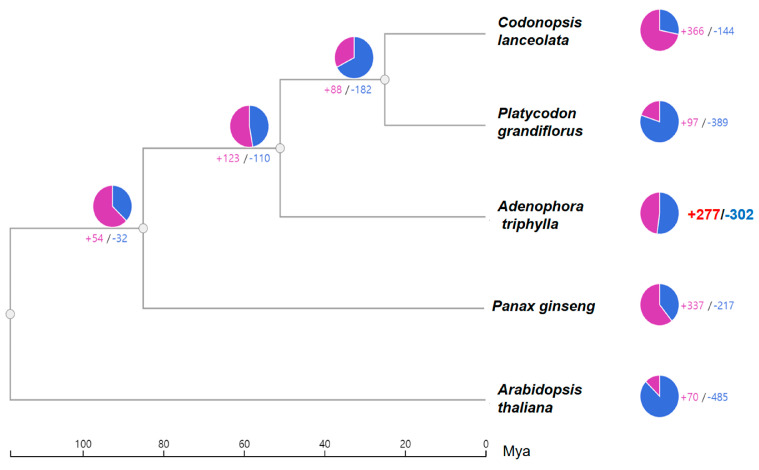
Estimation of gene family expansion and contraction on each evolutionary branch among *Adenophora triphylla*, *Codonopsis lanceolata*, *Platycodon grandiflorus*, *Panax ginseng*, and *Arabidopsis thaliana*. The numbers of expanded and contracted gene families are shown at the nodes in the phylogenetic tree. Magenta indicates the number of expanded gene families, whereas the blue number denotes contractions. Pie charts on the right show the proportions of these categories.

To investigate the evolutionary divergence of *A. triphylla,* expansion and contraction analyses of the gene family were performed between *A. triphylla* and four plant species. During divergence of the five plant species, *A. triphylla* was separated from the rest approximately 51–118 million years ago. Evolutionary divergence analysis revealed that 277 expanded and 302 contracted gene families clustered in *A. triphylla* (Figure 3). The clustered gene family was identified as an abundant GO term using GO enrichment analysis. Among the 277 genes that expanded during evolution between the groups, the GO terms were related to protein phosphorylation and protein serine/threonine kinase activity. Among these, protein phosphorylation is the most important activity in almost all cellular processes [35], and serine/threonine protein kinases play a role in apoptosis [36]. Among the 302 genes that contracted during evolution between the groups, GO terms were related to protein ubiquitination and oxidoreductase activity (Supplementary Table S13). Among these, protein ubiquitination is a post-translational modification process with many cellular functions [37], and oxidoreductases are a family of enzymes that catalyze redox reactions [38].

### 2.5. β-Amyrin Synthase Genes in A. triphylla

A total of 70 genes were identified to be related to triterpenoid saponin biosynthesis in *A. triphylla* using the KEGG database (Supplementary Table S14). Triterpenoid saponins play an important role in determining the quality of medicinal plants belonging to the Campanulaceae family [39], and *bASs* are important oxidosqualene cyclases involved in triterpenoid saponin biosynthesis [25]. Therefore, the identified 22 *bAS* genes among the 70 genes involved in triterpenoid saponin synthesis provide evidence of their major contribution to triterpenoid saponin modifications in *A. triphylla*. To identify the *bAS* genes commonly found in the Campanulaceae family, comparisons were made between *A. triphylla*, *P. grandiflorus*, and *C. lanceolata*. A total of 22, 14, and 13 genes were associated with β-amyrin production in *A. triphylla*, *P. grandiflorus*, and *C. lanceolata*, respectively (Supplementary Table S15). In particular, the *bAS* gene, consisting of 763 amino acids (NCBI/GenBank accession no., ASB17950.1), was identified in *A. triphylla* (Atj_C1300_0130T), *P. grandiflorus* (Pg_chr04_12440T), and *C. lanceolata* (Cl_chr02_36110T). This gene may be involved in the key synthase responsible for β-amyrin compound synthesis in the Campanulaceae family. Additionally, 22 *bAS* genes suggest the potential for species-specific saponin synthesis in *A. triphylla*. Triterpenoid saponins, such as platycodin D in *P. grandifloras* [9], lancemaside A in *C. lanceolate* [40], and dammarenediol-II in *P. ginseng* [41], have specifically evolved for individual plant species. The presence of an expanded *bAS* genes in *A. triphylla* suggests the possibility of discovering novel triterpenoid saponins in this plant.

### 2.6. Analysis of Transcriptomes Involved in the Development of Different Leaf Types

Leaves of *A. triphylla* interestingly show different morphological characteristics even within the same species [17]. Leaves of *A. triphylla* were round (elliptic or ovate) when the plant first germinated from seeds, but linear (lanceolate or cuneate) leaves appeared after it germinated from the bulb in the second year (Supplementary Figure S2). To explore the biological processes involved in the two leaf types, RNA sequencing was performed on two types of *A. triphylla* leaves, including nine round types (AT1-round) and nine linear types (AT2-linear) (Supplementary Figure S2 and Supplementary Table S17). After decontaminating the raw reads, we obtained 6,798,070,576–7,078,472,389 clean reads from 18 samples. Then, the reads were mapped to the *A. triphylla* genome. An average of 47,641,622 reads from 18 samples were used for genome mapping, with an average mapping rate of 88.5% (Supplementary Table S17).

**Figure 4:**
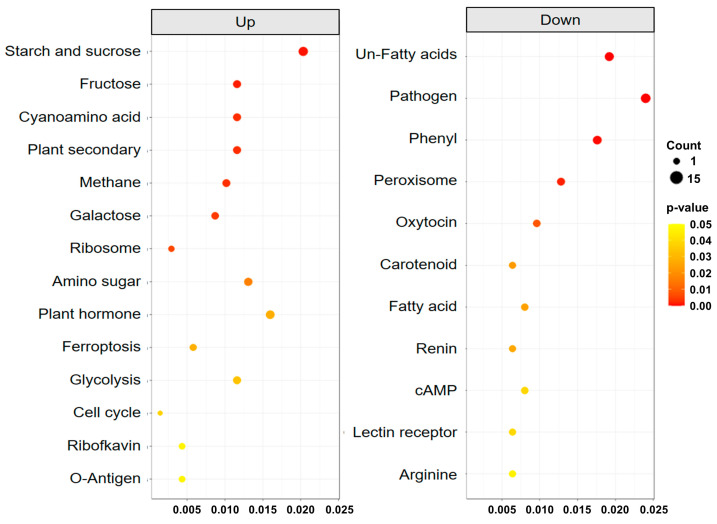
Scatter plot of Kyoto Encyclopedia of Genes and Genomes enrichment analysis between round and linear leaf types. The *x*-axis shows the ratio of differential to total genes, and the *y*-axis the enriched biological pathways. Dot size represents the positive correlation with the number of differentially expressed genes in the pathway, and color indicates the *p*-value.

To reveal the physiological responses in the two leaf types, we evaluated the biological pathways using GO and KEGG databases. Differentially expressed gene (DEG) analysis between AT1-round and AT2-linear showed a total of 1314 defined genes, including 689 upregulated and 625 downregulated genes (Supplementary Figure S3). KEGG functional enrichment analyses using the identified 1314 genes showed 14 significantly enriched pathways in the upregulated gene group (*p* < 0.05) and 11 in the downregulated gene group. The most enriched upregulated pathway was starch and sucrose metabolism, whereas the most downregulated pathway was a biosynthesis pathway involving unsaturated fatty acids (Figure 4).

In the GO analysis, both upregulated (Supplementary Figure S4) and downregulated gene groups (Supplementary Figure S5) were mainly enriched in oxidoreductase activity in the molecular function category. However, for oxidoreductase activity, the upregulated genes act on the sulfur group of donors (GO:0016667), whereas the downregulated genes act on paired donors with oxidation (GO:0016717). In the time interval between the development of the two leaf types, AT1-round was mainly enriched in response to heat (GO:0009408) and the endoplasmic reticulum pathway related to plant stress [42] (Supplementary Figure S6), and AT2-linear was enriched in pyrimidine metabolism pathways related to cellular development [43] (Supplementary Figure S7). Various metabolic differences at the point where the leaf originates are likely to cause different developmental patterns of leaves. In tomato (*Solanum lycopersicum*), the leaf initiation point can be determined by spatial differential regulation of carbohydrate metabolism in the apical meristem, which is associated with the spiral phyllotaxy pattern [44]. In addition, phytohormone biosynthetic mutants of brassinosteroid exhibit variations in leaf shape when compared to the wild type [44], while differences in auxin activity are also closely associated with leaf shape and development [15]. These results suggest that imbalances in phytohormone synthesis within plant tissues may contribute to the development of different leaf types. Therefore, the two types of leaves in *A. triphylla* are likely caused by differences in carbohydrate and phytohormone metabolism between seeds and bulbs.

## 3. Materials and Methods

### 3.1. Plant Materials and DNA Preparation

*A. triphylla* (Japanese lady bell) was collected from the National Institute of Horticultural and Herbal Science in Eumseong, Korea, and registered at the National Agrobiodiversity Center under voucher number IT272706. High molecular weight genomic DNA was extracted from young leaves using a Qiagen Cell Culture DNA Maxi Kit (Qiagen, Germantown, MD, USA). Quality of the genomic DNA was examined using a NanoDrop 2000 (Thermo Fisher Scientific, Santa Clara, CA, USA) and Agilent 2200 TapeStation (Agilent Technologies, Santa Clara, CA, USA).

### 3.2. Library Preparation, Sequencing, and Genome Size Estimation

Illumina and Nanopore sequencing libraries were prepared using a TruSeq DNA PCR-free kit (Illumina, San Diego, CA, USA) and an ONT 1D ligation sequencing kit (Oxford Nanopore Technologies, Oxford, UK), respectively. Sequencing was performed using an Illumina HiSeqX platform (Illumina) and 1D flow cells (Oxford Nanopore Technologies). After sequencing data trimming, Illumina PE (Phred score > 20) and Nanopore sequencing data (Q ≥ 7) were trimmed using the Trimmomatic [45] and Porechop [46], respectively.

The genome size of *A. triphylla* was estimated via k-mer frequency analyses of the trimmed Illumina PE data using Jellyfish (ver. 2.0 with optimal k-mer value of 17) [47] and GenomeScope (k-mer value of 23) [48].

### 3.3. Genome Assembly

Two assembly pipelines were used to assemble the *A. triphylla* genome. Pipeline 1 was composed of Canu [49], SMARTdenovo [50], Pilon [51], NextPolish [52], and Purge_haplotig [53], while Pipeline 2 was composed of NextDenovo [54], NextPolish [52], and Purge_haplotig [53] (Supplementary Table S3). For Pipeline 1, the trimmed Nanopore sequence data were self-corrected using the Canu assembler and the corrected sequences assembled de novo using SMARTdenovo with a minimum read length of 1000 bp. The assembled contig sequences were polished using Pilon with trimmed PE data. In Pipeline 2, the trimmed Nanopore data were assembled de novo using the NextDenovo assembler and the assembled contig sequences polished using NextPolish with the trimmed Nanopore and PE sequence data. Haplotigs (duplicate copy contig of the same region by heterozygosity) were removed using Purge_haplotig because they could interfere with downstream processes and cause erroneous results such as linking of alleles. Genome assembly quality assessed using the LAI [29] value and BUSCO [55] score with the embryophyta_odb10 library. The coverage of Illumina PE reads in the assembled genome was analyzed using BWA-MEM [56].

### 3.4. Gene Annotation

Protein-coding regions and transcriptome sequences were predicted in the assembled genome sequence using well-known annotation tools, such as Maker3 (https://www.yandell-lab.org/software/index.html, accessed on 14 December 2023), Snap, Augustus, GeneMark-ES, and EvidenceModeler (Supplementary Figure S8). The balloon flower [8,9], sun flower [57], Korean ginseng [58], and *A. thaliana* [34] sequences were used for the annotation of protein-coding genes. For transcriptome evidence, *A. triphylla* and *A. polyantha* sequences (NCBI accession no., SRR11994226) were used. A total of 195,939 transcripts were prepared, with a total length of 171.7 Mb. Genome sequences were masked with consensus repeat sequences, which were identified and characterized using RepeatModeler and RepeatMasker programs [59]. Finally, to improve annotation quality, we selected gene sequences for which the annotation evidence distance (AED) score was less than 1 which indicates there is no evidence to support the annotation, as in previous studies [25,60].

For functional annotation of protein-coding genes, all predicted proteins were aligned against those found in the NCBI non-redundant protein database using the Diamond tool with an e-value cutoff of 1 × 10^−5^ [61]. The aligned proteins were annotated as the best-matched proteins. The annotated genes were classified using GO terms and assigned to a metabolic pathway using the KEGG database.

### 3.5. Genome Comparisons

Five plant species were used for genome comparison: *A. triphylla*, *C. lanceolata* (lance asiabell), *P. grandiflorus* (balloon flower), *P. ginseng* (Korean ginseng), and *A. thaliana*. Unique and shared gene sequences were clustered using the OrthoVenn3 web tool (ver. 4. 26.1) with an e-value cutoff of 1 × 10^−5^ [62]. The expansion and contraction of gene families were investigated using the CAFE5 program (ver. 2.1) [63] and OrthoVenn3 web tool. Divergence time information among the five plant species was retrieved from the Time-Tree database [64] and entered into OrthoVenn3 (*C. lanceolata* and *P. grandiflorus*: 25 million years ago (MYA), *A. triphylla* and *C. lanceolata*: 51 MYA, *A. triphylla* and *P. grandiflorus*: 51 MYA, *A. triphylla* and *P. ginseng*: 85 MYA, *A. triphylla* and *A. thaliana*: 118 MYA, *P. ginseng* and *A. thaliana*: 118 MYA).

### 3.6. RNA Sequencing and Transcriptome Analysis

*A. triphylla* accessions include leaf collections at different developmental stages of two leaf types and stress-treated samples (Supplementary Table S16). These samples were frozen in liquid nitrogen for RNA extraction. Total RNA was extracted using the RNeasy Mini Kit (Qiagen) and quantified using an Agilent 2100 Bioanalyzer (Agilent Technologies). Libraries were prepared with 2 μg of total RNA for each sample using a TruSeq Stranded mRNA LT Sample Prep kit (Illumina, San Diego, CA, USA). Then, indexed libraries were sequenced using an Illumina HiSeq X platform by Macrogen Incorporated (Seoul, Republic of Korea). The raw reads were preprocessed to remove low-quality, adapter sequences and bacterial sequences using Trimmomatic [45] and BBduk [65].

For transcriptome analysis, 18 samples (two leaf types, three replicates, and three time intervals) were selected from *A. triphylla* accessions in RDA GenBank (http://genebank.rda.go.kr/, accessed 8 October 2020), which were unstressed plant samples grown in a greenhouse (Supplementary Table S17). The processed reads were aligned to the *A. triphylla* genome using HISAT [66]. For expression profiling and DEG analysis, fragments per kilobase of transcript per million mapped reads were calculated based on the number of mapped RNA reads. The DESeq2 package was used to determine DEGs between samples [67]. The DEGs were evaluated with the up- and downregulated genes, and their biological roles were verified using GO enrichment and KEGG pathway database analyses. The *bAS*-related genes of triterpenoid saponin biosynthesis were identified using KEGG databases with eukaryote gene sets. The programs and parameter values used for transcriptome analysis were indicated in Supplementary Figure S9.

## 4. Conclusions

The Japanese lady bell (*A. triphylla*) is a popular medicinal plant in East Asia and Russia that has various therapeutic effects. This study is the first to report the genome size, genome assembly, and genomic characterization of *A. triphylla*. In the *A. triphylla* genome, 22 *bAS* genes were identified, providing indirect evidence for the biosynthesis of triterpenoid saponins in this plant. Analysis of GO and KEGG revealed differences in the activities of starch, sucrose, unsaturated fatty acid pathways, and oxidoreductase enzymes in the development of two leaf types. Our findings provide insights into *A. triphylla* genome evolution and could enable future genomics-aided breeding of important traits in the Campanulaceae family. These genomic resources are important for the improvement of agricultural traits for the use of *A. triphylla* as a source of pharmaceutical resources and health-related foods.

## Figures and Tables

**Table 1:** Genome assembly metrics and gene annotation statistics of *Adenophora triphylla*.

Parameter	Value
**Genome assembly (Pipeline 2)**	
Number of sequences	8432 contigs
Total length of sequences	2,477,772,497 bp
N50 length	403,696 bp
Smallest sequence	18,168 bp
Longest sequence	3,658,388 bp
Average length	293,853 bp
Complete BUSCO (version 5.0)	95.6%
**Gene annotation**	
Number of protein-coding genes	57,729
Total length of protein-coding genes	55,297,059 bp
Smallest gene length	102 bp
Longest gene length	16,275 bp
Average gene length	958 bp
Repeat content	79.2%
GC content	43.6%
Functionally annotated	88.4%
Complete BUSCO (version 5.0)	75.0%

BUSCO = Benchmarking Universal Single-Copy Orthologs score (embryophyta_odb10 library).

## Data Availability

All raw sequencing data, including RNA-Seq data, produced in this study have been deposited in the NCBI Sequence Rad Archive (SRA) under BioProject number PRJNA693119 and BioSample SAMN17377662. This whole genome shotgun project has been deposited in GenBank under accession number JAFEMH000000000 and BioProject number PRJNA693119. In addition, “The first highly contiguous genome assembly of Japanese lady bell *Adenophora triphylla*” files were uploaded to Figshare (https://doi.org/10.6084/m9.figshare.24084255, accessed on 5 September 2023).

## Supplementary Materials

The following supporting information can be downloaded at: https://www.mdpi.com/article/10.3390/genes15010058/s1. References [8,9,10,25,33,34,68,69,70] are cited in the supplementary materials.
