# Supplementary material for: First Contiguous Genome Assembly of Japanese Lady Bell (Adenophora triphylla) and Insights into Development of Different Leaf Types
Source: Genes (Basel). 2023 Dec 30;15(1):58. doi: 10.3390/genes15010058 (PMC10815912; doi:10.3390/genes15010058)
Supplement: Supplementary file 1 [file genes-15-00058-s001.zip › Supplementary_Figures_S.pdf]

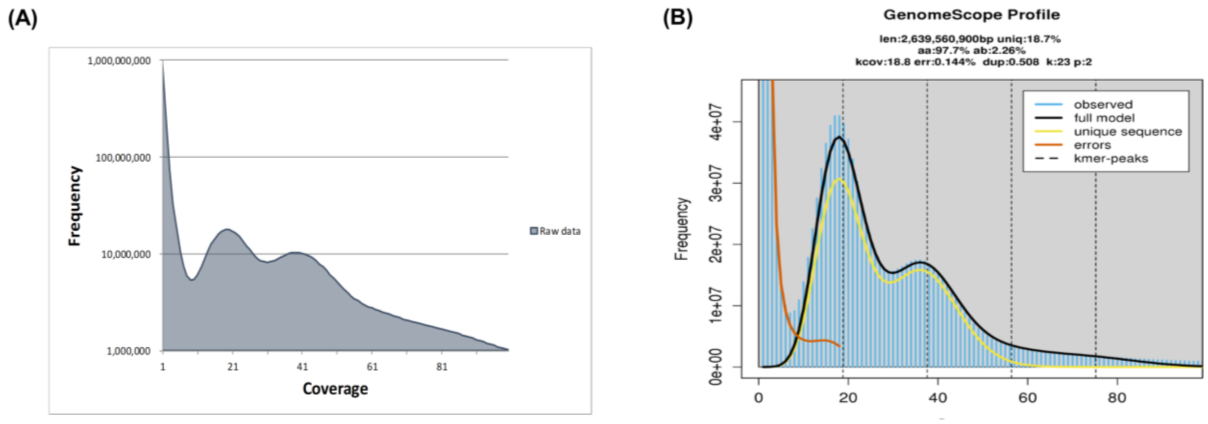

Supplementary Figure S1. Genome size estimation of *Adenophora triphylla*. (A) Genome size was estimated via k-mer frequency analysis using raw paired-end data (Jellyfish v 2.0 program with optimal k-mer value of 17). (B) Genome size was estimated using GenomeScope profile plots with a 23 k-mer value.

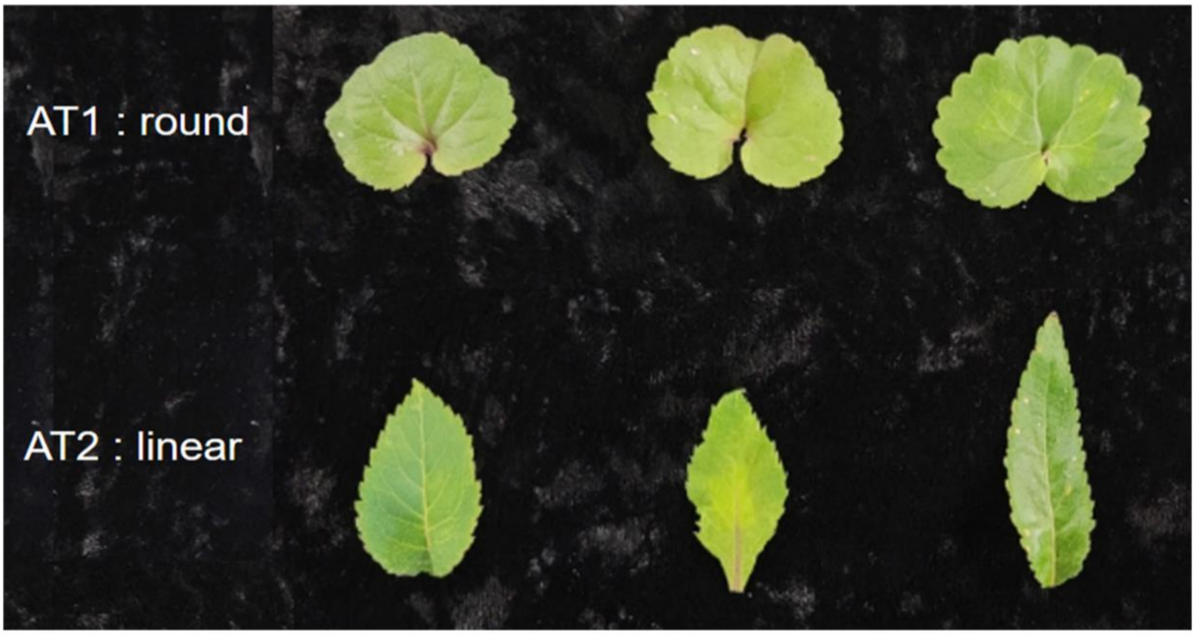

Supplementary Figure S2. Two leaf types (round and linear) of *Adenophora triphylla*.

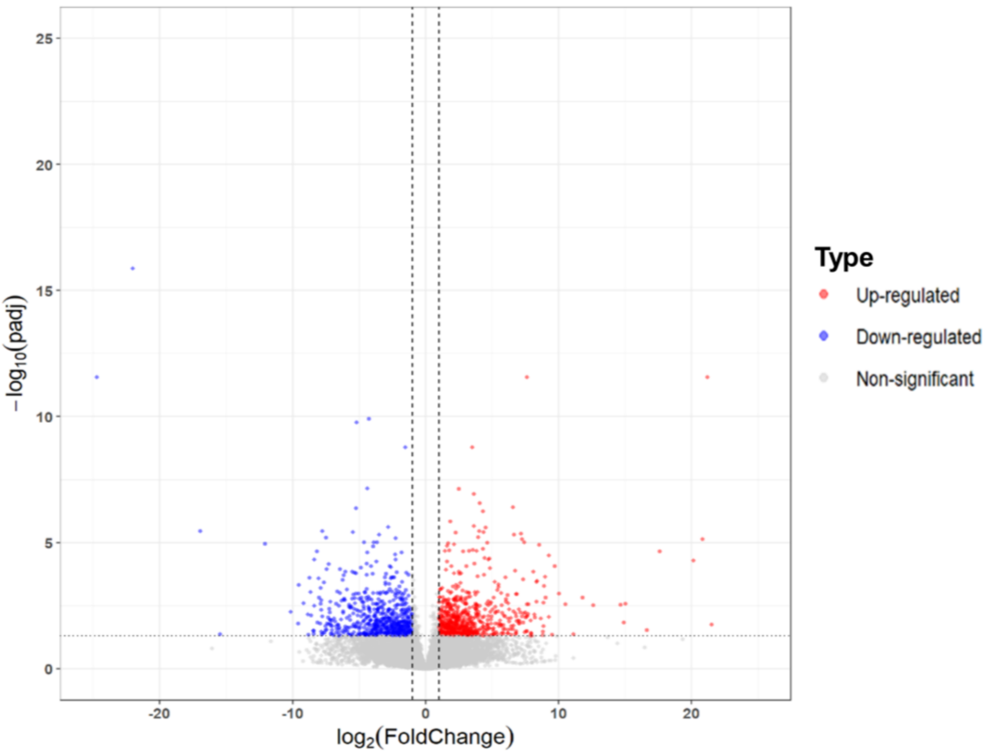

Supplementary Figure S3. Volcano plot between round (AT1-round) and linear (AT2-linear) leaf types. Red points indicate highly upregulated genes, whereas blue points represent the considerably downregulated genes.

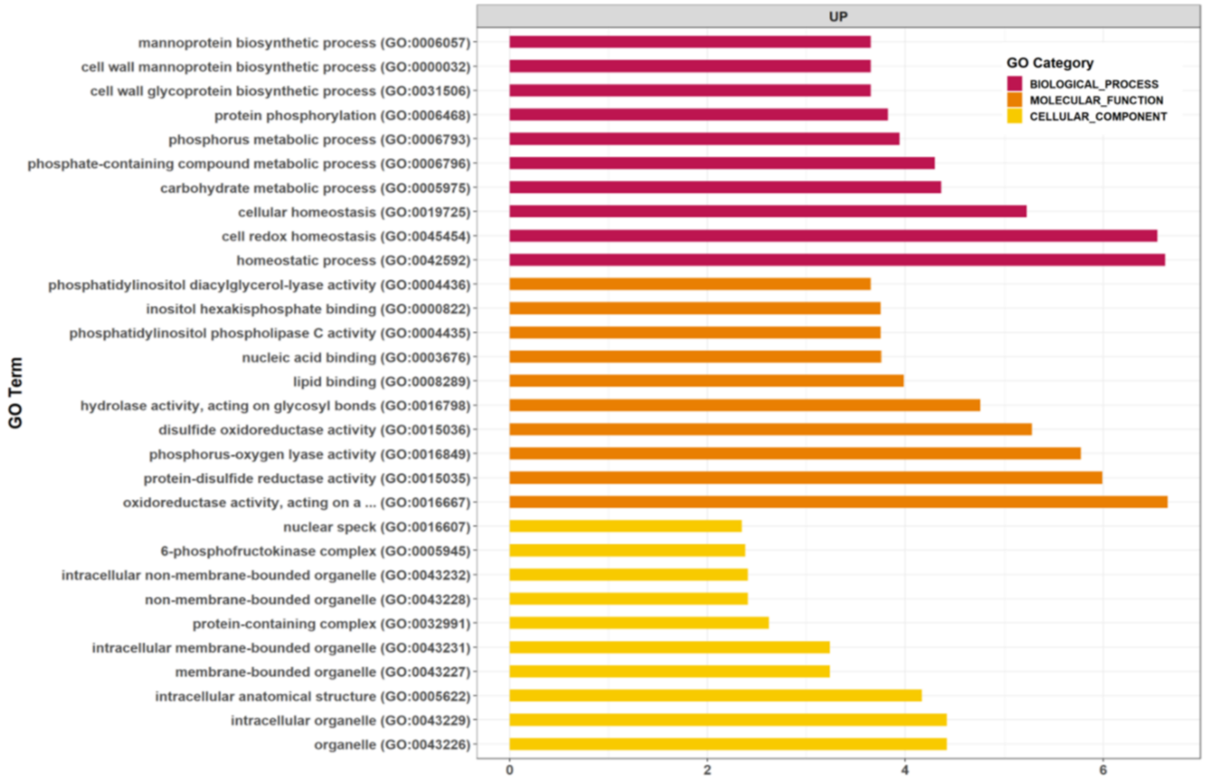

Supplementary Figure S4. Gene Ontology terms of upregulated genes between round (AT1-round) and linear (AT2-linear) leaf types.

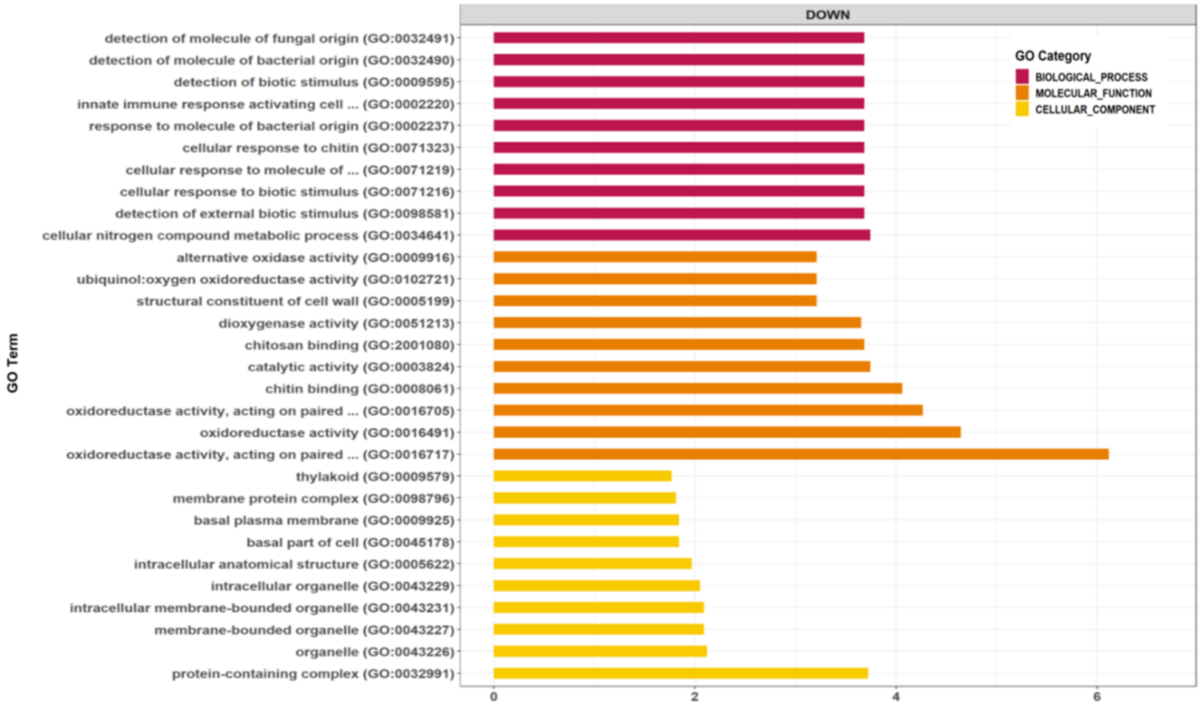

Supplementary Figure S5. Gene Ontology terms of downregulated genes between round (AT1-round) and linear (AT2-linear) leaf types.

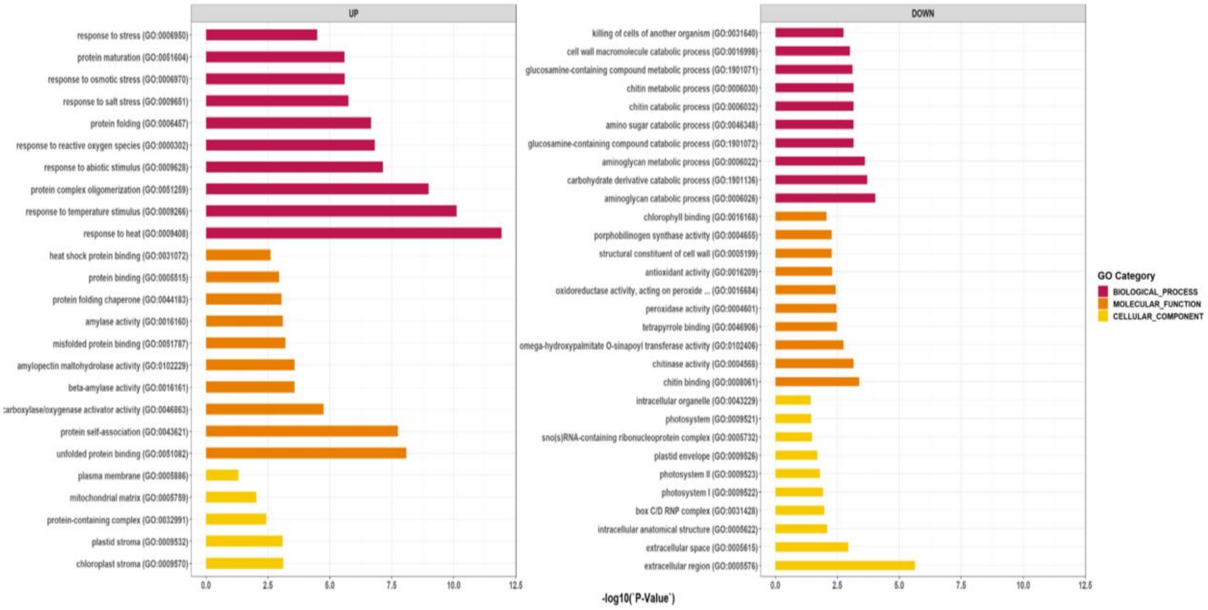

Supplementary Figure S6. The Gene Ontology (GO) terms of AT1-round between time intervals (75 and 90 DAG) were compared with 60 DAG (**Supplementary Table S17**). The GO terms between 60 and 75 DAG (left figure). The GO terms between 60 and 90 DAG (right figure). DAG, day after germination.

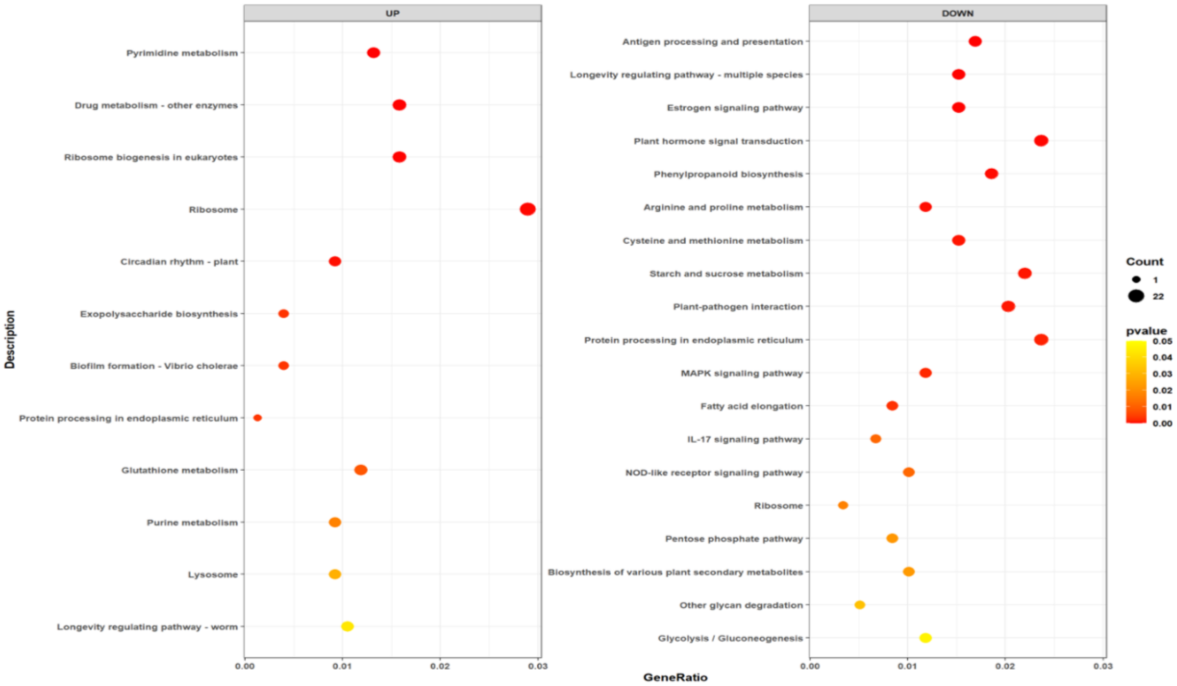

Supplementary Figure S7. The Kyoto Encyclopedia of Genes and Genomes (KEGG) pathways of AT2-linear between time intervals (75 and 90 DAG) were compared with 60 DAG (**Supplementary Table S17**). The KEGG pathways between 60 and 75 DAG (left figure). The KEGG pathways between 60 and 90 DAG (right figure). The X-axis shows the ratio of differentially expressed genes (DEGs) to the background number in a particular pathway. Size of the dots represents the number of genes, and the color the range of the p-value. DAG, day after germination.

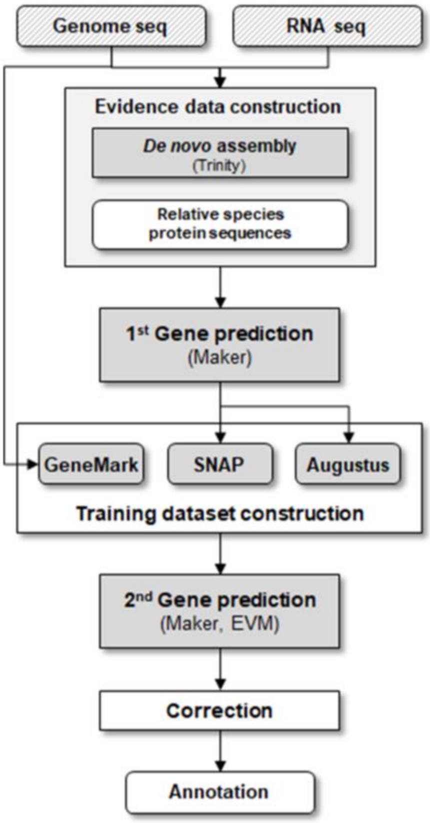

Supplementary Figure S8. Annotation pipeline for gene prediction from the genome sequence of *Adenophora triphylla*. Trinity (version 2.8.4) (Grabherr et al., 2011), MAKER3 (version 3.01.03) (https://www.yandell-lab.org/software/maker.html), SNAP (version 2006-07-28) (Zaharia et al., 2011), AUGUSTUS (version 3.3.2) (Stanke et al., 2006), GeneMark-ES (version 4.38), and EvidenceModeler (version 1.1.1) were used for this analysis with default parameters.

Procedure	Program	Version	Options
Preprocessing	Trimmomatic	0.39	LEADING:3, TRAILING:3, SLIDINGWINDOW:4:20, MINLEN:50
	BBDuK	38.87	K=31, mcf=0.5
Expression profiling	HISAT2	2.2.1	--dta-cufflinks
	HTSeq	2.0.1	-S no
	DESeq2	1.36.0	Default
	ggplot2	3.3.6	NA
Functional annotation	DIAMOND	2.1.6	blastp max-target-seqs:20 evalue:1e-5
	InterProScan	5.57-90.0	Default
	BLAST2GO CLI	1.4.4	Default
	BLAST2GO	5.2.5	Default
	KAAS(web tool)	-	SBH method

Supplementary Figure S9. Transcriptome pipeline for preprocessing, expression profiling, and functional annotation of *Adenophora triphylla*.
